# Neurodegeneration in Alzheimer's disease: caspases and synaptic element interdependence

**DOI:** 10.1186/1750-1326-4-27

**Published:** 2009-06-26

**Authors:** Dale E Bredesen

**Affiliations:** 1 Buck Institute for Age Research, 8001 Redwood Blvd., Novato, CA USA 94945; 2 Department of Neurology, University of California, San Francisco, CA USA 94143

## Abstract

Extensive genetic, biochemical, and histological evidence has implicated the amyloid-β peptide (Aβ) in Alzheimer's disease pathogenesis, and several mechanisms have been suggested, such as metal binding, reactive oxygen species production, and membrane pore formation. However, recent evidence argues for an additional role for signaling mediated by the amyloid precursor protein, APP, in part via the caspase cleavage of APP at aspartate 664. Here we review the effects and implications of this cleavage event, and propose a model of Alzheimer's disease that focuses on the critical nature of this cleavage and its downstream effects.

## Review: programmed cell death, cell death signaling, and neurodegenerative disease

Many of the diseases that affect the nervous system feature an abnormality of cell death of one sort or another: for example, developmental and neoplastic disorders of the nervous system feature dysregulation of the intrinsic cellular programs that mediate cell death. Such dysregulation may also occur in neurodegenerative, infectious, traumatic, ischemic, metabolic, and demyelinating disorders. Therefore, targeting the central biochemical controls of cell survival and death may potentially represent a productive therapeutic approach. Furthermore, recent results from stem cell studies suggest that the fate of neural stem cells may also play an important role in disease outcomes, and therefore cell death apparently plays a central role in many neurological diseases, and potentially in their prevention and treatment.

Early studies of neuronal survival focused on the status of external factors such as pH, glucose availability, and the partial pressure of oxygen. While these are clearly critical determinants, research over the past few decades has revealed a more active, and more plastic, role for the cell in its own life/death decision than was previously appreciated. Complementing this concept, studies of the internal suicide programs of neural cells have offered new potential targets for therapeutic development.

In neurodegenerative diseases such as Alzheimer's disease, neurons in various nuclei are lost in disease-specific distributions. However, the neuronal loss is a relatively late event, typically following synaptic dysfunction, synaptic loss, neurite retraction, and the appearance of other abnormalities such as axonal transport defects. This progression argues that cell death programs may play at best only a secondary role in the neurodegenerative process. However, emerging evidence from numerous laboratories has suggested an alternative possibility: that although cell death itself occurs late in the degenerative process, the pathways involved in cell death *signaling *do indeed play critical roles in neurodegeneration, both in sub-apoptotic events such as synapse loss and in the ultimate neuronal loss itself [1-4].

Although initial comparisons of the intrinsic suicide program in genetically tractable organisms such as the nematode *C. elegans *failed to disclose obvious relationships to genes associated with human neurodegenerative diseases – e.g., presenilin-1 and the β-amyloid precursor protein (APP) do not bear an obvious relationship to any of the major *C. elegans *cell death genes (ced-3, ced-4, or ced-9) – more recent studies have begun to disclose a fundamental relationship between developmental and degenerative processes [1,4-8]. For example, Nikolaev and Tessier-Lavigne found that trophic factor withdrawal from developing neurons results in neurite retraction that is mediated by a cleavage product of sAPPβ [1]. A detailed understanding of the interrelationship between fundamental cell death programs and neurodegenerative processes is still evolving, and it promises to offer novel approaches to the treatment of these diseases.

## Caspases: activation and role in programmed cell death

**Figure 1:**
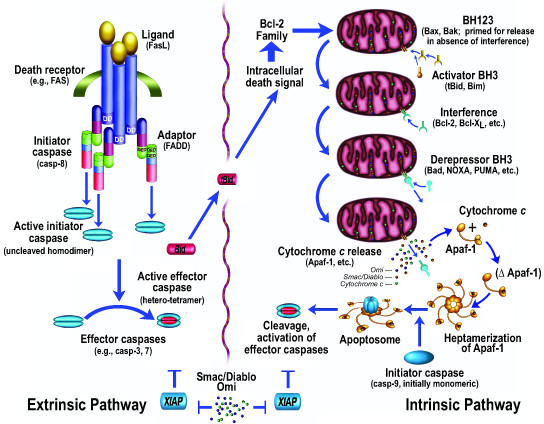
**Programmed cell death pathways may be divided generally into intrinsic and extrinsic pathways**.

Apoptosis (Fig. 1) has been studied extensively, with over 100,000 papers published on the subject . Morphologically, cells typically round up, form blebs, undergo zeiosis (an appearance of boiling), chromatin condensation, nuclear fragmentation, and the budding off of apoptotic bodies. Phosphatidylserine, normally placed asymmetrically such that it faces internally rather than externally on the plasma membrane (due to a flipase that flips the phosphatidlyserine so that it faces internally), appears externally during apoptosis [9]. These morphological and histochemical changes are largely the result of the activation of a set of cell-suicide cysteine proteases referred to as caspases (Table 1) [10,11]. The characteristics of these proteases are described more fully below.

**Table 1:** Comparison of the apoptotic caspases.

**Caspase**	**Group**	**Cleavage Preference^1^**	**Prodomain**	**Comments**
Caspase-2	Initiator	DAVAD	CARD	
Caspase-3	Executioner	DEVD	Short	
Caspase-6	Executioner	VEVD	Short	Implicated in neurite retraction [4]
Caspase-7	Executioner	DEVD	Short	
Caspase-8	Initiator	IETD	DED	Extrinsic pathway
Caspase-9	Initiator	LEHD	CARD	Intrinsic pathway
Caspase-10	Initiator	(similar to caspase-8)	DED	Extrinsic pathway

1. Note that preferences do not exclude cleavage at other sites with Asp in the P1 position, and that the P1' position is also important (see text).

The biochemical activation of apoptosis occurs through two general pathways (Fig. 1): the intrinsic pathway, which is mediated by the mitochondrial release of cytochrome c and resultant activation of caspase-9; and the extrinsic pathway, originating from the activation of cell surface death receptors such as Fas, resulting in the activation of caspase-8 or -10 [12]. A third general pathway, which is essentially a second intrinsic pathway, originates from the endoplasmic reticulum and also results in the activation of caspase-9 [13-17]. In addition, other organelles, such as the nucleus and Golgi apparatus, also display damage sensors that link to apoptotic pathways [18]. Thus, damage to any of several different cellular organelles may lead to the activation of the apoptotic pathway.

Activation of the intrinsic pathway of apoptosis (e.g., by DNA damage) leads to cytochrome c release from the mitochondria, and the released cytochrome c interacts with a cytosolic protein, Apaf-1, via the WD-40 repeats of Apaf-1, leading to the exposure of a (d)ATP-binding site on Apaf-1, which, when occupied, induces a conformational change resulting in heptamerization. The resultant exposure of the Apaf-1 CARD (caspase activation and recruitment domain) recruits caspase-9 into this apoptosomal complex, and the resulting induced proximity of caspase-9 molecules leads to their activation [19]. Activation of the apical caspase-9 leads to a cascade of caspase activation, including the downstream, effector caspases, such as caspase-3 and caspase-7. However, the active caspases-3, 7, and 9 can be held in check by the IAP (inhibitor of apoptosis) proteins, such as XIAP [20], which may function as both direct inhibitors of caspase activity (in the case of caspase-9, by inhibiting dimerization) and as E3 ligases that mediate caspase degradation by the proteasome [21]. This IAP-mediated block may itself be released by additional mitochondrially-derived proteins, Smac/DIABLO [22,23] and Omi/HtrA2 [24,25]. Smac ("second mitochondrial activator of apoptosis"), for example, binds to IAP proteins, preventing their inhibition of caspases, thus allowing caspase activation despite the presence of the otherwise inhibitory IAP proteins.

As opposed to the intrinsic pathway, which utilizes caspase-9 as its apical caspase, the extrinsic pathway utilizes caspase-8 or caspase-10. In the best characterized example, Fas is bound by trimeric Fas ligand, resulting in the interaction of an intracytoplasmic domain of Fas, dubbed the death domain, with a similar death domain in an adaptor molecule, FADD (Fas-associated death domain protein). FADD displays, in addition to its death domain, another domain called a DED (death effector domain), and this domain interacts with a similar DED domain in caspase-8 [26]. The induced proximity of the apical caspase again leads to activation, as is the case for caspase-9. Also as for caspase-9, the initial caspase activation allows this upstream caspase to attack downstream, effector pro-caspases (somewhat analogous to what occurs in the thrombotic cascade, except that cysteine aspartyl-specific proteases (caspases) are utilized instead of serine proteases), cleaving and activating the effector caspases such as caspase-3 and caspase-7. In addition, FLIP(L) (FLICE-like inhibitory protein, long form), previously considered an inhibitor of extrinsic pathway activation, may act as a caspase-8 activator by functioning as a preferable dimeric partner of caspase-8 (over caspase-8 itself), resulting in activation by heterodimerization of what would otherwise be activated less readily by homodimerization [27].

Both the intrinsic and extrinsic pathways of apoptosis thus converge on the activation of effector caspases by initiator caspases. Caspases are cysteine aspartyl-specific proteases that cleave with remarkable specificity at a small subset of aspartic acid residues. Their substrates, the number of which is unknown but probably somewhere between 0.5% and 5% of proteins, contribute to the apoptotic phenotype in several different ways: for example, following cleavage, their substrates contribute to proteolytic cascade activation, cellular structural alterations, inactivation of repair mechanisms (e.g., DNA repair), internucleosomal DNA cleavage, phagocytic uptake signaling, mitochondrial permeabilization, and other effects. Although the substrates of caspases represent a small minority of the overall proteomic makeup, in neurodegenerative diseases in general, and in Alzheimer's disease in particular, these substrates are over-represented: APP (and its related family members APLP1 and APLP2), the presenilins, and tau are all caspase substrates [28-32].

Caspases are synthesized as zymogens, but differ markedly in their activation: the initiator caspases (caspase-8, -9, and -10) exist as intracytoplasmic monomers until dimerization is effected by adaptor molecules, such as FADD. Contrary to earlier assumptions, cleavage of apical caspases is neither required nor sufficient for activation [33]. The zymogenicity – i.e., the ratio of activity of the active form to that of the zymogen – of these caspases is relatively low, in the range of 10–100 [33], and thus the (monomeric) zymogens themselves are actually somewhat active. These caspases display relatively large prodomains that are utilized in the protein-protein interactions that mediate activation – CARD (caspase activation and recruitment domain) in caspase-9, and DED (death effector domain) in caspase-8 and -10. The substrates of the initiator caspases typically display I/L/V-E-X-D in the P4-P1 positions (with cleavage just carboxyterminal to the P1 residue), with preference for small or aromatic residues in the P1' position [33].

The apical caspases activate effector caspases such as caspase-3 and -7. In contrast to the apical caspases, the effector caspases exist as dimers within the cell, display high zymogenicity (greater than 10,000 for caspase-3) and short prodomains, and are activated by cleavage rather than induced proximity. Cleavage produces a tetramer with two large subunits of 17–20 kilodaltons and two small subunits of 10–12 kilodaltons. Because of a difference in the S4 pocket (which interacts with the P4 residue on the substrate) structure of these caspases (in comparison to the apical caspases), with similarity in the S1 and S3 pockets, their substrate preference is D-E-X-D, with a two orders of magnitude preference for Asp over Glu in the P4 position [33].

Caspases that do not fit squarely within these two groups include caspase-2, which displays a long prodomain like an apical caspase but has a substrate preference more similar to effector caspases (with the exception that, unlike other caspases, it also has a P5 preference (for small hydrophobic residues)); caspase-6, which has a short prodomain like effector caspases yet a substrate preference similar to apical caspases; and the inflammatory caspases (-1, -4, -5) involved in the processing of interleukin-1β and interleukin-18. These latter are thought not to play a role in pcd; however, inhibition in some paradigms such as cerebral ischemia has indeed been associated with a reduction in infarct size [34].

Caspase-12 is anomalous: in the murine system, it appears to play a role in apoptosis induced by endoplasmic reticulum (ER) stress [15,17,35]. However, murine caspase-12 lacks Arg341, which in other caspases is critical for the Asp specificity in the P1 position [33], and instead features a Lys in this position. Nonetheless, proteolytic activity has been reported for caspase-12 [17], catalytically inactive caspase-12 inhibits ER stress-induced apoptosis [15], caspase-uncleavable caspase-12 also inhibits ER stress-induced apoptosis, and mice null for caspase-12 are less susceptible to amyloid-β toxicity than wild type mice [35]. In the great majority of humans, however, a nonsense mutation is present in the caspase-12 gene, preventing expression of an active caspase [36]. Those without such a mutation are at increased risk for sepsis, due to the attenuation of the immune response to endotoxins such as lipopolysaccharide [37].

## Caspase activation in neurodegeneration: association or requirement?

Evidence for caspase activation in neurodegeneration has been derived both from the use of antibodies directed against newly-exposed proteolysis-dependent epitopes (neo-epitopes) generated by caspase cleavage [4,6,38] and from the inhibition of neurodegeneration by caspase inhibitors [39,40]. One of the critical goals for dissecting the relationship between pcd and neurodegeneration is to determine the specificity of the trigger: specifically, is neurodegeneration the result of an imbalance in physiological signaling events (analogous to what occurs in neoplasia) or, as more commonly suggested, the result of a relatively nonspecific toxic effect of a peptide or protein aggregate? If the latter, then secondary neurodegeneration may occur due to loss of trophic support, excitotoxicity, or any number of other secondary effects. If the former, then specific, physiologically-relevant transduction events that underlie neurite retraction and synapse loss may potentially be triggered directly by neurodegeneration-associated transcriptional and post-transcriptional events. In other words, is neurodegeneration analogous to cancer in being an imbalance in physiological signals (not from oncogenes and tumor suppressor genes, but those that mediate synaptic maintenance and synaptic re-organization)? Evidence on both sides exists: for example, numerous toxic properties have been attributed to the Aβ peptide, such as reactive oxygen species generation and metal binding, among others [41]. However, signal transduction effects have also been attributed to Aβ peptide, such as binding and multimerization of amyloid precursor protein, with resultant complex formation and direct caspase activation [42].

Since the neurodegenerative process may be induced by widely varying insults – from misfolded proteins to reactive oxygen species to caspase recruitment complexes, as well as other mechanisms – and yet produce a relatively small number of syndromes, the existence of a death network is suggested. The putative network may be entered from many different sites, but once triggered, would follow similar interdependent biochemical pathways, with little dependence on the point of entry. This notion is compatible with the findings that therapeutics aimed at different pathways (caspase activation, mitochondrial release of cytochrome c, metal binding, reactive oxygen species scavenging, etc.) all exert partially salutary effects. However, it also suggests that a complete halt of the neurodegenerative process may require therapeutics that address all of the network's interacting pathways.

## Trophic factors and cellular dependence in Alzheimer's disease

Neurons, as well as other cells, depend for their survival on stimulation that is mediated by various receptors and sensors, and pcd may be induced in response to the withdrawal of trophic factors, hormonal support, electrical activity, extracellular matrix support, or other trophic stimuli [43]. For years it was generally assumed that cells dying as a result of the withdrawal of required stimuli did so because of the loss of a positive survival signal, for example mediated by receptor tyrosine kinases [44]. While such positive survival signals are clearly extremely important, data obtained over the past 15 years argue for a complementary effect that is pro-apoptotic, activated by trophic stimulus withdrawal, and mediated by specific receptors dubbed "dependence receptors" [45,46]. Over a dozen such receptors have now been identified, and examples include DCC (deleted in colorectal cancer), Unc5H2 (uncoordinated gene 5 homologue 2), neogenin, RET, Ptc, and APP [46-50]; [51-55]. These receptors interact in their intracytoplasmic domains with caspases, including apical caspases such as caspase-9, and may therefore serve as sites of induced proximity and activation of these caspases. Caspase activation leads in turn to receptor cleavage, producing pro-apoptotic fragments [48,56]; however, mutation of the caspase cleavage sites of dependence receptors suppresses pcd mediated by the receptors [45,48]. A striking example of this effect was obtained in studies of neural tube development: withdrawal of Sonic hedgehog from the developing chick spinal cord led to apoptosis mediated by its receptor, Patched, preventing spinal cord development; however, transfection of a caspase-uncleavable mutant of Patched blocked apoptosis and restored significant development, even in the absence of Sonic hedgehog [57].

Thus cellular dependence on specific signals for survival is mediated, at least in part, by specific dependence receptors that induce apoptosis in the absence of the required stimulus – when unoccupied by a trophic ligand, or when bound by a competing, anti-trophic ligand – but block apoptosis following binding to their respective ligands [43,46,49]. Expression of these dependence receptors therefore creates cellular states of dependence on the associated trophic ligands. These states of dependence are not absolute, since they can be blocked downstream in some cases by the expression of anti-apoptotic genes such as bcl-2 or p35 [43,47,58]; however, they result in a shift of the apostat [12,59] toward an increased likelihood of triggering apoptosis. In the aggregate, these receptors may serve as a molecular integration system for trophic signals, analogous to the electrical integration system comprised of the dendritic arbors within the nervous system.

Cellular dependence on trophic signals was originally described in the developing nervous system, but neurodegeneration may utilize the same pathways: the β-amyloid precursor protein (APP) exhibits several features characteristic of dependence receptors, including an intracytoplasmic caspase cleavage site (Asp664) [31,32], co-immunoprecipitation with an apical caspase (caspase-8), caspase activation, derivative pro-apoptic peptides (see below), and suppression of apoptosis induction by mutation of the caspase cleavage site. [31,42].

These findings raise several questions: first, does the caspase cleavage of APP occur in human brain, and, if so, is this increased in patients with Alzheimer's disease? Second, if this cleavage is prevented, is the Alzheimer's phenotype affected? Third, is there a physiological role for this cleavage event? These questions are addressed below.

## Alzheimer's disease: an imbalance in cellular dependence?

Extensive genetic and biochemical data have implicated the Aβ peptide as a central mediator of Alzheimer's disease, but the mechanism(s) of action remains controversial: some have emphasized the ability of Aβ to generate a sulfuranyl radical involving methionine 35, others have focused on the metal-binding property of Aβ, others have pointed to its aggregating property, and still others have implicated its detergent-like effects on some membranes, just to list a few of the mechanisms proposed [41]. These proposed mechanisms share a focus on the chemical and physical properties of the Aβ peptide. However, cellular signaling is emerging as a complementary mechanism by which Aβ exerts its critical effects, and multiple candidates have surfaced as key downstream mediators, including APP itself, the insulin receptor, and tau, among others [42,60,61]. These cellular signals may also mediate neuronal dependence on trophic support, as described below.

**Figure 2:**
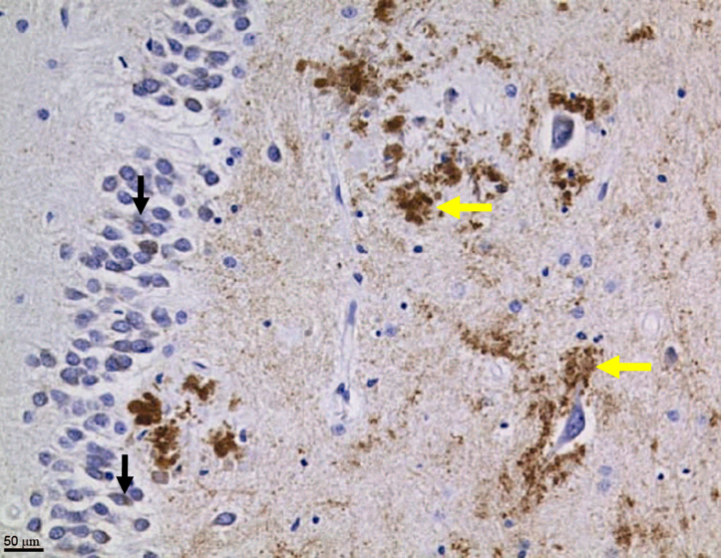
**Caspase cleavage of APP, indicated by immunohistochemical detection of the APP664 neo-epitope, in the brain of a patient with Alzheimer's disease**. Note perinuclear cytoplasmic staining in the granular cell layer of the hippocampus (black vertical arrows) as well as intense, apparently extracellular plaque-like deposits (yellow horizontal arrows).

Neo-epitope antibodies directed against residues 657–664 of human APP disclosed the presence of caspase-cleaved APP fragments in human brain (Fig. 2), especially in the hippocampal region [7], with an approximately four-fold increase in Alzheimer's patients over age-matched controls. However, in brains without Alzheimer's pathology, there was an inverse relationship between age and immunohistochemical detection of APPneo, with a different distribution than in AD brains: whereas, in the Alzheimer brains, the staining was primarily in somata, in the non-Alzheimer brains, the staining was observed predominantly in the processes. These findings suggest that the caspase cleavage of APP occurs physiologically and is reduced with age, but that this process remains more active in association with Alzheimer's disease.

The effect of preventing the caspase cleavage of APP on the Alzheimer's phenotype was evaluated in AD model transgenic mice that express APP with Swedish and Indiana mutations that are associated with familial Alzheimer's disease. Although the caspase mutation (D664A) had no effect on plaque formation, or on the production of Aβ peptides 1–40 or 1–42, the D664A mutation prevented the synapse loss, early p21-activated kinase (PAK) phosphorylation, dentate gyral atrophy, electrophysiological abnormalities (including reductions in excitatory post-synaptic potentials (EPSPs) and long-term potentiation (LTP)), neophobia, and memory deficits that characterize Alzheimer model mice [2,4,62]. These findings indicate that key features of the Alzheimer's phenotype, at least in a standard transgenic mouse model, depend on the presence of the caspase cleavage site within APP. Yet, as noted above, extensive previous work has shown that the phenotype is critically dependent on Aβ, suggesting that the APP caspase site may lie downstream from the Aβ accumulation that is unaffected by the D664A mutation [42,61]. This possibility has received support from studies showing that Aβ interacts directly with APP in the Aβ region itself, leading to multimerization, caspase cleavage, and cell death signaling [42,61].

If APP does indeed function as a dependence receptor, Alzheimer's disease may be considered a "state of altered dependence" (Appendix 1). What then is/are the trophic ligand(s) for APP? Several candidate APP interactors have been described, such as collagen (types I and IV), heparan sulfate proteoglycan, laminin, glypican, and F-spondin [63-65]. In the case of F-spondin's interaction with APP, β-secretase activity is reduced. Lourenco et al. have recently shown that netrin-1, a multifunctional axon guidance and trophic factor, also binds APP [8]. Furthermore, netrin-1 also interacts with Aβ itself, and thus Aβ may interfere with netrin-1 binding to APP. The binding of netrin-1 to APP results in enhanced interaction of APP with Fe65 and Dab, up-regulation of KAI1, and a marked reduction of net Aβ production [8].

These findings suggest a model in which the Aβ peptide functions as an anti-trophin, blocking netrin's guidance and trophic effects, binding and oligomerizing APP, recruiting and activating caspases, engendering the processing of APP at Asp664, and inducing neurite retraction, and, ultimately, neuronal cell death [4,42,61,66]. Whether the D664A mutation of APP exerts effects beyond the prevention of caspase cleavage (e.g., an alteration of the intracytoplasmic structure of APP) is not yet known. However, irrespective of the mechanism, the results suggest that APP signal transduction may be important in mediating Alzheimer's disease [67], at least in the transgenic mouse model, possibly downstream from Aβ oligomerization and binding of APP.

The results obtained in the transgenic mouse model of AD also suggest an alternative to the classic models of AD. As noted above, chemical and physical properties of Aβ have been cited as the proximate cause of AD pathophysiology. However, these theories do not explain why Aβ is produced ubiquitously and constitutively, nor do they offer a physiological function for the Aβ peptide, or account for the improvement in AD model mice that occurs with a reduction in tau protein [60].

**Figure 3:**
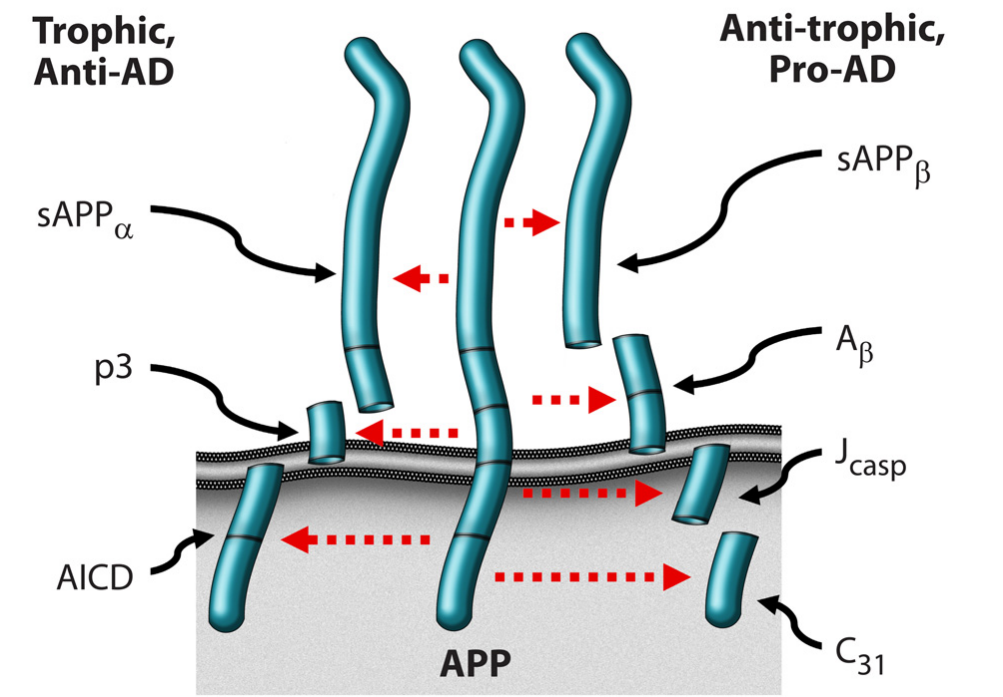
**Alternative cleavage of APP to produce four peptides that mediate synaptic loss, neurite retraction, and ultimately programmed cell death ("the four horsemen"); or three peptides that mediate synaptic maintenance and inhibit programmed cell death ("the wholly trinity")**. Among the factors that mediate the decision between these two pathways are included trophic effects such as netrin-1 and anti-trophic effects such as Aβ peptide.

**Figure 4:**
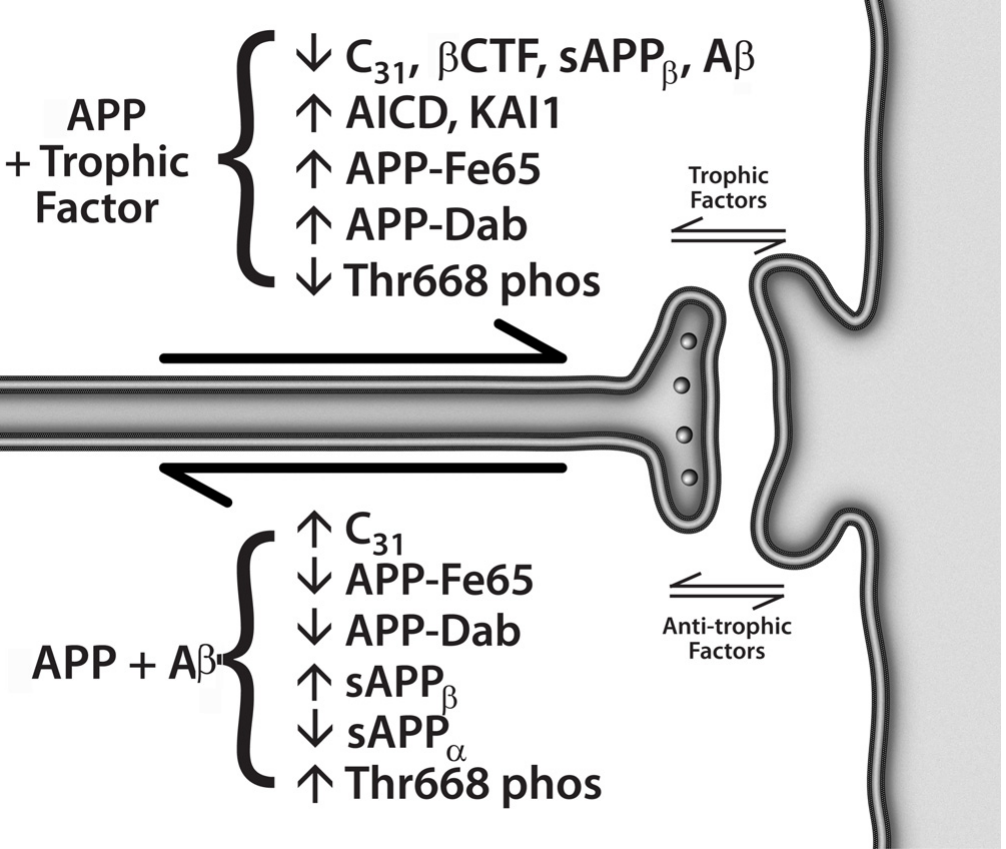
**Synaptic element interdependence model of synaptic maintenance, reorganization, and Alzheimer's disease**. The pre-synaptic and post-synaptic elements are interdependent, and provide both trophic influences (e.g., neurotrophins, netrin-1, laminin, collagen, and synaptic activity itself) and anti-trophic influences (e.g., amyloid-β peptide). Trophic support leads to the processing of APP into three peptides that support synaptic maintenance, whereas the withdrawal of trophic support leads to alternative processing, to four peptides that mediate synaptic inhibition, synaptic loss, neurite retraction, and ultimately, programmed cell death. In this model, the Aβ peptide functions as an anti-trophin, and, since it leads to APP processing that produces additional Aβ peptide, it is "prionic", i.e., Aβ begets additional Aβ.

An alternative model, presented in Figs. 3 and 4, argues that APP is indeed a dependence receptor, and that it functions normally as a molecular switch in *synaptic element interdependence*: in this model, both the pre-synaptic element and the post-synaptic element are dependent on trophic support, including soluble factors such as netrin, substrate molecules such as laminin, neurotransmitters, and neuronal activity, as well as other factors. In the presence of adequate trophic support, APP is cleaved at the alpha and gamma sites, generating three peptides – sAPPα, p3, and AICD – that support cell survival and synaptic maintenance. However, a reduction in trophic support alters the processing of APP, reducing the α/β ratio of cleavage, and leading to the production of four peptides – sAPPβ, Aβ, Jcasp, and C31 – that mediate a reduction in synaptic transmission, synaptic loss, neurite retraction and, ultimately, programmed cell death [1,4,8,31,62]. In this model, Alzheimer's disease is suggested to be an imbalance in physiological signaling pathways that mediate synaptic maintenance vs. synaptic re-organization, mediated at least in part by APP, functioning in synaptic element interdependence, as part of a plasticity module that includes other receptors such as the common neurotrophin receptor, p75^NTR ^and the axon guidance receptor DCC, among others [68] (see Appendix 2 regarding proposed follow-up studies). It is important to note that multiple groups have described a pro-apoptotic effect of AICD, in contrast to the model proposed here (e.g., [69,70]); however, since AICD can be cleaved at Asp664 to give rise to two pro-apoptotic peptides – Jcasp and C31 – it is critical to evaluate AICD with a mutation that prevents this caspase cleavage, and previous studies have not included these data. Thus the pro-apoptotic effect attributed to AICD may be due to its ability to give rise to Jcasp and C31.

## Conclusion

We present here a model for Alzheimer's disease that is based not on chemically and physically-mediated toxic effects of Aβ, but on imbalanced signal transduction. The model suggests that the imbalance lies in the ratio of the signals that mediate synaptic maintenance, neurite extension, and cell survival vs. those that mediate synaptic reorganization, neurite retraction, and programmed cell death – essentially, memory retention vs. forgetting and memory reorganization. This model suggests that Aβ has a physiological function as a neuromodulatory peptide, and at least in some cases that it functions as an anti-trophin – competing, for example, with netrin-1 for binding to APP. Whether it has analogous functions related to its described interactions with other receptors, such as PrP, p75^NTR^, and RAGE, remains to be determined. This model also proposes that synaptic element interdependence is a critical factor in synaptic maintenance vs. reorganization, with associated effects on memory retention vs. loss. Both pre-synaptic and post-synaptic elements exert trophic and anti-trophic influences on each other, and the balance determines whether synaptic maintenance or reorganization will occur. The molecular details of the model are summarized in Fig. 4.

## Abbreviations

(d)ATP-binding: deoxyadenosine triphosphate binding; (D664A): aspartate residue 664 mutated to alanine; AD: Alzheimer's disease; AICD: amyloid precursor protein intracytoplasmic domain; APP: amyloid precursor protein; Aβ: amyloid-β peptide; Apaf-1: apoptosis activating factor-1; APLP1: APP-like protein 1; APLP2: APP-like protein 2; Arg341: arginine residue 341; Asp: aspartic acid; Asp664: aspartic acid at residue 664 (of APP, based on APP695 numbering); Bcl-2: B-cell lymphoma gene 2; C31: carboxyterminal 31 residues; CARD: caspase activation and recruitment domain; Ced-3: cell death gene 3; ced-4: cell death gene 4; ced-9: cell death gene 9; Dab: disabled protein; DCC: deleted in colorectal cancer; DED: death effector domain; DNA: deoxyribonucleic acid; E3 ligases: E3-ubiquitin protein ligases; ER: endoplasmic reticulum; EPSP: excitatory post-synaptic potential; FADD: Fas-associated death domain protein; FLIP(L): FLICE-like inhibitory protein, long form; Glu: glutamate; I/L/V-E-X-D: isoleucine/leucine/valine-glutamate-any amino acid-aspartate; IAP: inhibitor of apoptosis protein; Jcasp: juxtamembrane fragment of APP produced by caspase cleavage and gamma-secretase cleavage; KAI1: Kangai 1 (suppression of tumorigenicity 6, prostate; CD82 antigen (R2 leukocyte antigen, antigen detected by monoclonal and antibody IA4)); LTP: long-term potentiation; Lys: lysine; Neo-epitopes: newly-exposed proteolysis-dependent epitopes; Omi/HtrA2: mitochondrial serine protease that antagonizes IAP proteins; PAK: p21-activated kinase; p3: peptide of approximately three kilodaltons, derived from APP by cleavage at the alpha-secretase site and the gamma-secretase site; p35: protein of approximately 35 kilodaltons that inhibits caspases; derived from baculovirus; P4 position: fourth amino acid aminoterminal to the cleavage site of a given protease; p75^NTR^: common neurotrophin receptor; pcd: programmed cell death; Ptc: patched protein; RET: rearranged during transfection; S1 and S3 pockets: pockets of the protease that interact with the P1 and P3 residues of the substrate, respectively; sAPPα: soluble fragment of APP derived from the cleavage by alpha-secretase; sAPPβ: soluble fragment of APP derived from the cleavage by beta-secretase; Smac/DIABLO: second mitochondrial activator of apoptosis protein/DIABLO protein; Unc5H2: uncoordinated gene 5 homologue 2; WD-40 domain: protein binding motif that contains ~7 regions ~40 amino acids long containing a conserved tryptophan and aspartic acid; XIAP: inhibitor of apoptosis protein X.

## Competing interests

The author declares that they have no competing interests.

## Appendix 1: Key Observations

• Caspase cleavage may be critical in both apoptotic and sub-apoptotic events (e.g., synapse loss) in neurodegenerative disease.

• APP exhibits the characteristics of a dependence receptor.

• APP may be cleaved in two alternative patterns: to produce four peptides that mediate synaptic loss, neurite retraction, and ultimately programmed cell death ("the four horsemen"); or three peptides that mediate synaptic maintenance and inhibit programmed cell death ("the wholly trinity"). Among the factors that mediate the decision between these two pathways are included trophic effects such as netrin-1 and anti-trophic effects such as Ab peptide.

• A model of Alzheimer's disease is presented that is based on synaptic element interdependence, imbalanced signal transduction, and caspase activation. In this model, the amyloid-beta peptide functions as an anti-trophin and exhibits "prionic" positive feedback.

## Appendix 2: Critical Next Steps

• Evaluate transgenic mice producing alternative peptides: the "four horsemen" or the "wholly trinity".

• Establish the structural basis of the interactions between APP and netrin-1; and between APP and the amyloid-beta peptide.

• Characterize the signaling network that mediates the neurite retractive, pro-Alzheimer's phenotype vs. the synaptic maintenance, anti-Alzheimer's phenotype.
